# Women with Metabolic Syndrome and Unhealthy Lifestyle Factors Are at a Higher Risk for Hyperuricemia

**DOI:** 10.3390/jcm12227159

**Published:** 2023-11-18

**Authors:** Seonyoung Kang, Kyungdo Han, Jinhyoung Jung, Yeonghee Eun, In Young Kim, Eun-Mi Koh, Seulkee Lee, Hoon-Suk Cha, Hyungjin Kim, Jaejoon Lee

**Affiliations:** 1 Department of Medicine, Samsung Medical Center, Sungkyunkwan University School of Medicine, Seoul 06355, Republic of Korea; seonyoung.kang@samsung.com (S.K.); seulkeelee.lee@samsung.com (S.L.); hoonsuk.cha@samsung.com (H.-S.C.); 2 Department of Statistics and Actuarial Science, Soongsil University, Seoul 06978, Republic of Korea; hkd917@naver.com; 3 Department of Medical Statistics, College of Medicine, Catholic University of Korea, Seoul 06591, Republic of Korea; jungjin115@naver.com; 4 Division of Rheumatology, Department of Internal Medicine, Kangbuk Samsung Hospital, Sungkyunkwan University School of Medicine, Seoul 06351, Republic of Korea; yeonghee.eun@samsung.com; 5 Department of Medicine, National Police Hospital, Seoul 05715, Republic of Korea; ariatansi@naver.com; 6 Korean Health Insurance Review and Assessment Service, Seoul 06653, Republic of Korea; emkoh@skku.edu; 7 Department of Medical Humanities, Samsung Medical Center, Sungkyunkwan University School of Medicine, Seoul 06351, Republic of Korea

**Keywords:** hyperuricemia, metabolic syndrome, lifestyle behaviors

## Abstract

Hyperuricemia (HUA) has become a significant medical concern due to its complications and links to metabolic syndrome (MetS) and cardiovascular disease (CVD), which result in increased mortality. The pathogenic processes associated with unhealthy behaviors, MetS, and HUA can be cooperative and potentially synergistic in the activation of risk factors. Recent research has shown sex-based differences in the relationship between HUA and its associated risk factors. This study aimed to investigate these differences, particularly in the context of MetS and CVD risk factors and unhealthy lifestyles. We also aimed to evaluate the joint effects of these factors based on sex. We conducted a cross-sectional study using nationally representative survey data from the Korean National Health and Nutritional Examination Survey 2016–2018. We performed multivariable logistic regression analysis, calculating adjusted odds ratios (ORs) with their 95% confidence intervals (CIs). We also conducted subgroup analyses based on sex and the presence of MetS with or without unhealthy lifestyle factors (tobacco use, alcohol intake). We found sex-based differences in the relationships between HUA and MetS, CVD risk factors, and lifestyle behaviors. Our major finding was a significant association between MetS and HUA in both men and women, regardless of alcohol consumption and smoking status, and this association was stronger in women. We also observed a synergistic effect of MetS and lifestyle factors on the risk of HUA, particularly in women, in whom the risk of HUA increased up to four times compared to the reference group. A sex-based clinical strategy for HUA is necessary to reduce related complications and their socio-economic burden.

## 1. Introduction

Hyperuricemia (HUA) plays significant roles in gout development and urinary stone formation. Also, accumulating evidence suggests that HUA may be an indicative marker of, or play a role in, the pathogenesis of various diseases, including metabolic, cardiovascular, and renal disease [1,2,3]. The presence of HUA is related to increased mortality and is affected by various lifestyle and behavior variables [4,5,6,7,8,9,10,11,12,13]. Given the increased prevalence of HUA and its clinical impact, understanding its risk factors has become increasingly important.

HUA is a condition that exhibits differences in disease characteristics, risk profiles, and associated complications between sexes. Additionally, some studies have proposed examining sex not just for differences but treating it as a significant biological variable [14]. Thoroughly analyzing sex-specific factors when studying certain diseases in order to gain insights into clinical profiles and develop effective preventive strategies is crucial. There is some evidence for sex-based differences in the interrelationship between the risk of HUA and MetS. In most studies, an association between HUA and MetS has been found in both genders [15,16,17,18,19,20,21]. However, in studies related to lifestyle factors associated with HUA, particularly concerning smoking and alcohol consumption, the results regarding gender-specific associations varied across different studies [5,6,11,22,23]. Since smoking and alcohol consumption are linked to MetS [24], it is necessary to examine the influence of these lifestyle factors on their association with HUA, considering the presence or absence of MetS.

However, previous research studies had methodological limitations because they did not perform such subgroup or stratified analyses. Additionally, it is crucial to assess the risk of HUA in situations where individuals have both MetS and unhealthy lifestyle factors, as this scenario is common. Previous studies, however, mainly focused on understanding the association between HUA and a single specific factor. Second, other potential confounding variables such as BMI [22] and CKD [11,22], known to be associated with the risk of HUA, were not considered. Third, the largest-scale study in Asia, which examined metabolic comorbidities and lifestyle factors associated with HUA, was carried out in China with 11,567 participants. However, it had limitations as it only included participants aged 35 or older, and a similar restriction was observed in another Chinese study, which focused on participants aged 40 or older. These age-based limitations may affect the generalizability of the findings [22,23].

Evaluating the interactive effects of risk factors can provide valuable information for identifying at-risk individuals in preventive and therapeutic settings.

Kim IY et al. reported that in women with both MetS and obesity, the risk of HUA significantly increases compared to men [25]. A British study found that an unhealthy lifestyle, which includes smoking, alcohol consumption, poor dietary habits, and physical inactivity, along with a genetic predisposition for gout and cardiometabolic diseases, increases the risk of gout over sevenfold [26]. In an Indonesian study, overweight smokers had a ninefold increased risk of gout compared to non-smokers with normal weight [27].

To our knowledge, there have been no studies evaluating the risk of HUA in individuals who simultaneously have both MetS and unhealthy lifestyle factors (smoking, alcohol use) in Korea.

Taking into account the limitations of previous studies, we conducted a comprehensive analysis using data from the Korean National Health and Nutritional Examination Survey (KNHANES) from 2016 to 2018. Our primary objective was to clarify the complex relationship between MetS, major cardiovascular risk factors, unhealthy lifestyles, and HUA. We also aimed to consider the interactive effects of these factors regarding sex disparities, using a larger dataset and more adjusted variables than in previous studies. The purpose of this study was to identify high-risk groups associated with HUA to provide information for the development of preventive strategies for HUA.

## 2. Materials and Methods

### 2.1. Study Population and Data Source

The KNHANES is a cross-sectional, nationwide survey in Korea that evaluated the health and nutritional status of the population. The KNHANES survey material consists of interviews, physical examinations, and laboratory tests. KNHANES uses a complex, multistage probability design to provide a nationally representative sample of the non-institutionalized civilian Korean population [28]. The present study included participants 19 years or older with data on serum urate levels using data collected from the 2016 through 2018 annual KNHANES cycles. Of the 24,269 participants in the 2016–2018 KNHANES, individuals under 19 years of age and those with missing variables of interest were excluded. As a result, data from a total of 16,288 subjects, 7154 males and 9134 females, were included in the analysis.

### 2.2. Collection of Data

Demographic, socio-economic, and health-related behavior data were collected and included age, sex, education level, household income, alcohol consumption, smoking frequency, physical activity, and dietary habits. The prevalence of comorbidities such as hypertension (HTN), diabetes mellitus (DM), and hyperlipidemia was included. Trained staff measured height, body weight, waist circumference (WC), and blood pressure (BP) using standardized instruments. Body mass index (BMI) was calculated by dividing the weight in kilograms by height in meters squared. Laboratory tests such as fasting glucose, lipid profile, creatinine level, and uric acid level were performed in the survey. Potential associated factors were selected on the basis of previous studies and clinical experiences [29].

### 2.3. Definition of Variables

HUA was defined as a serum uric acid level ≥ 7.0 mg/dL in men and ≥ 6.0 mg/dL in women [30]. MetS was defined as the presence of at least three of the five criteria established by the revised National Cholesterol Education Program Adult Treatment Panel III (NECP-ATPIII), and we adopted an Asian-specific WC threshold proposed by the International Diabetes Foundation (IDF) [31,32,33]. These criteria were (1) central obesity: WC ≥ 90 cm in men and ≥ 85 cm in women; (2) elevated BP: systolic BP ≥ 130 mmHg and/or diastolic BP ≥ 85 mmHg or on antihypertensive medications; (3) hyperglycemia: elevated FPG ≥ 100 mg/dL or on anti-diabetic medications; (4) hypertriglyceridemia: elevated triglycerides (TG) ≥ 150 mg/dL or on medication for elevated TG; and (5) reduced high-density lipoprotein cholesterol (HDL-C): HDL-C < 40 mg/dL in men and <50 mg/dL in women or on medication for reduced HDL-C. Alcohol consumption was classified into three groups: non-drinker, mild drinker (<30 g/day), and heavy drinker (≥30 g/day). Physically active was defined by WHO recommendations, more than 150 min of moderate-intensity aerobic physical activity throughout the week or more than 75 min of vigorous-intensity aerobic physical activity throughout the week or an equivalent combination of moderate- and vigorous-intensity activity. HTN was defined as a systolic BP > 140 mmHg, a diastolic BP > 90 mmHg, a previous diagnosis of HTN by a physician, or being prescribed an anti-hypertensive medication. DM was defined as a fasting blood glucose level > 126 mg/dL or by a previous history of DM or by taking hypoglycemic medication or insulin. Hyperlipidemia was defined as a fasting total C ≥ 240 mg/dL or by prescription of lipid-lowering medication. Chronic kidney disease (CKD) was defined as an eGFR < 60 mL/min/1.73 m^2^.

### 2.4. Statistical Analysis

Categorical variables are presented as numbers and percentages (%), and continuous variables are presented as mean values ± standard deviation (SD). Student’s *t*-test and chi-square test were used to compare baseline characteristics. Multivariable logistic regression analysis was performed to assess the association between HUA and MetS, cardiometabolic disease, and unhealthy behaviors. We calculated the adjusted odds ratio (OR) and its 95% confidential interval. All statistical analyses were conducted using the SAS survey procedure (version 9.4; SAS institute, Cary, NC, USA).

### 2.5. Ethics Statement

All procedures in each KNHANES were approved by the Institutional Review Board of Korean Centers for Disease Control and Prevention (KCDC), and written informed consent was obtained from participants. The present study protocol followed the ethical guidelines of the 1975 Declaration of Helsinki as revised in 1983. The Institutional Review Board of Samsung Medical Center approved the current study (number: SMC 2023-04-050).

## 3. Results

### 3.1. Baseline Characteristics of the Study Population

Among 16,288 participants enrolled in this study, there were 7154 males and 9134 females. Table 1 presents the baseline characteristics of the study population according to sex. The mean uric acid level was 5.95 mg/dL in men and 4.41 mg/dL in women. The proportion of HUA was higher in men than in women. Low education level, low income, low physical activity, hyperlipidemia, and dining alone were observed more frequently in women than in men. The male participants in the study had a significantly high proportion of individuals who engaged in heavy alcohol consumption and had an elevated smoking habit frequency. In men, the occurrence of comorbid conditions such as CKD, obesity, and MetS components other than hyperlipidemia was more prevalent when compared to women. The proportion of women subjects who indicated their progression into menopause was approximately 40%.

### 3.2. Association between MetS, Cardiovascular Risk Factors, and Lifestyle Components and HUA

Current smoking was associated with greater odds of HUA during this period among women (OR 1.60, CI 1.01–2.53) in an adjusted model. In contrast, there was no relationship between smoking status and HUA among men. An association between alcohol consumption and HUA was confirmed in both sexes. Significant associations were found in men with heavy alcohol intake and in women with low alcohol intake after adjustment for confounding factors. There was no significant association of HUA with physically active exercise for both sexes (Table 2).

Table 3 shows that both men and women displayed a significant association between MetS and HUA. Women had a higher associated risk between HUA and MetS than men (OR 2.40 CI 1.78–3.23 in women and OR 1.80 CI 1.47–2.20 in men). All metabolic components were associated with HUA among women in the adjusted model. In men, hypertriglyceridemia, high BP, and low HDL were associated with HUA. Metabolic components had a dose-dependent association with HUA in both sexes but especially in women.

**Figure 1:**
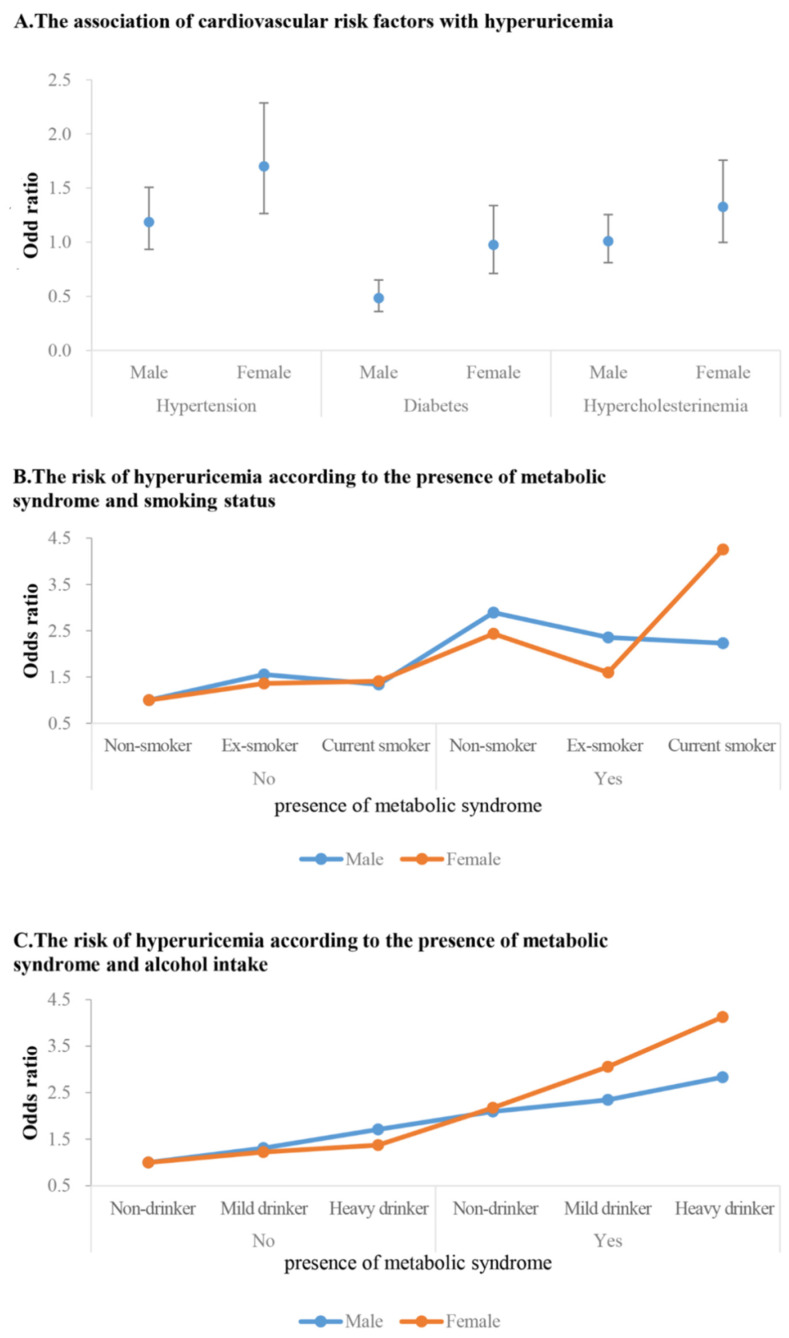
The risk of hyperuricemia related to cardiovascular risk factors and according to MetS and lifestyle factors subgroup.

Figure 1A and Table 4 show the association of major cardiovascular risk factors with the risk of HUA. HTN was significantly associated with increased risk of HUA in women (OR 1.7, 95% CI 1.27–2.29). This was not the case with men. In men, subjects with DM were associated with decreased risk of HUA (OR 0.48, 95% CI 0.36–0.65).

### 3.3. Subgroup Analysis

Figure 1B and Table 5 show the adjusted ORs (95% CI) for having HUA according to the presence of MetS and/or smoking status between sexes. Subjects without both factors were assigned to a reference group. MetS was associated with an increased risk of HUA in both men and women regardless of smoking status. However, in women with MetS, current smoking status was associated with an increased risk of HUA, and particularly in women with MetS who were current smokers, the risk of developing HUA was four times higher (OR 4.26, 95% CI 2.47–7.35). Among men without MetS, only ex-smoker men were associated with increased OR of HUA compared to reference groups (OR 1.56, 95% CI 1.01–2.21). In men with MetS, non-smokers were associated with a higher OR compared to ex-smokers or current smokers (OR 2.89, 95% CI 1.93–4.33).

Figure 1C and Table 6 show the adjusted ORs (95% CI) for having HUA according to the presence of MetS and alcohol intake in subgroup analysis. MetS was associated with a higher risk of HUA in both sexes regardless of alcohol intake. In the case of heavy-drinking men, an association with the risk of hyperuricemia was observed regardless of the presence of MetS. However, when MetS was concurrent, the risk further increased. In women, there was no significant impact of alcohol consumption on HUA when MetS was not present. In subjects with MetS, alcohol intake quantity increases were associated with increased OR for HUA. Notably, the risk was substantially amplified among women who had MetS and were heavy drinkers. These women were at four times greater risk of HUA than women without both conditions.

## 4. Discussion

Previous studies on factors related to HUA have already confirmed the different risk profiles and associations between genders. In the case of MetS, an association with HUA has been consistently observed in both men and women across various studies. However, regarding lifestyle factors related to HUA, such as smoking and alcohol consumption, previous findings regarding gender-specific associations are not consistent [5,6,22].

Since smoking and alcohol consumption are related to MetS, it is necessary to examine the influence of these lifestyle factors on their association with HUA, taking into account the presence or absence of MetS. However, previous research has methodological limitations as the studies did not perform such subgroup or stratified analyses. Additionally, there has been a lack of evaluation regarding the potential synergistic effect on the risk of HUA when unhealthy lifestyle factors and MetS coexist.

In this study, the relationships between HUA and MetS, cardiovascular risk factors, and unhealthy lifestyles were examined based on sex. Consistent with previous findings [6], a greater prevalence of HUA was observed among males compared to females. We found sex-based differences in the relationships between HUA and MetS, cardiovascular risk factors, and unhealthy lifestyles. We confirmed a significant correlation between MetS and HUA in both males and females, consistent with previous studies. However, this association was particularly prominent among women after controlling for other factors that may influence the findings. Furthermore, we examined the difference in the impact of unhealthy lifestyle factors, such as smoking and alcohol consumption, on HUA based on the presence of MetS. We observed that this difference varies according to gender. In males without MetS, ex-smokers and heavy drinkers showed an association with an increased risk of HUA. However, in cases where MetS coexisted, non-smokers and heavy drinkers exhibited the highest risk. On the other hand, in females without MetS, there was no significant association between unhealthy lifestyle factors, such as smoking and alcohol consumption, and the risk of HUA. However, when MetS was present, both smoking habits and alcohol consumption were associated with an increased risk of HUA, with a particularly noticeable increase in risk observed in current smokers and heavy drinkers. This risk was up to four times higher than that of the reference group. Briefly, we observed a synergistic effect of MetS and lifestyle factors on the risk of HUA, which showed different patterns depending on sex. Women who had MetS and either smoked or consumed alcohol excessively were highly susceptible to a high risk of HUA. This finding was, at least partially, in line with a recent study from the U.S. reporting that the combination of a strong genetic predisposition and overweight status or obesity results in an excess risk of incident gout that is greater than the sum of each exposure alone, especially among women [34]. The mechanisms of this possible interaction, particularly among women, remain to be clarified.

The inflammation and cell damage induced by these factors can lead to an increased release of cellular urate [35]. The observed vulnerability in women can be attributed to gender differences in the regulation of inflammation and MetS, along with the potential for more pronounced harmful effects of alcohol and smoking in women [36].

Physiologically, women have lower uric acid levels compared to men, but several findings have suggested a stronger association between HUA and women with certain risk factors [37,38]. A Chinese elderly community study showed that the relationship between HUA and MetS was more robust in women than in men [15]. In one prospective study, men with higher serum uric acid levels had a 1.6-fold higher risk of MetS, and women had more than a twofold higher risk [20]. In a group of obese individuals, the presence of inflammatory abnormalities, which are recognized as biomarkers for determining MetS, showed a stronger correlation with both uric acid and MetS in females [39]. This finding suggests that there may be sex-specific differences in the association of inflammation with these health conditions among obese individuals. A meta-analysis revealed a connection between HUA and the incidence of or mortality from CVD in women, while no such relationship was observed among men [40]. The present investigation revealed an association between the risk of HUA and MetS, which was observed in both sexes. However, this connection was found to be more pronounced in women. This finding was consistent with that of previous research. Moreover, the cumulative impact of MetS components on HUA exerted a greater influence on women compared to men. The underlying reason for these sex differences is complex and multifaceted. Potential explanations include sex hormonal effects or inherent variations in insulin sensitivity and body fat composition, factors that interact with HUA and MetS outcomes [41,42]. Additionally, lifestyle choices as well as social factors such as smoking and alcohol consumption may have differential effects on HUA depending on sex, an observation previously substantiated by studies cited here [11,23,43]. In a Mendelian randomization analysis conducted in Korea, a causal relationship between any alcohol consumption and HUA was found only in men [44]. However, in our study, an association was observed not only in men but also among women who consumed alcohol in moderation. Recent Chinese research has shown gender-specific differences in the impact of alcoholic beverage types on serum urate levels [45]. Therefore, future studies should consider the type of alcoholic beverage. 

Numerous studies have been conducted on the effects of smoking on urate based on sex, and the results are inconclusive. It is suggested that variations in research methodologies, the diversity of confounders, differences in smoking intensity, and limitations associated with self-reported lifestyle information may have played a role in this disparity [35]. Two recent Korean cohort studies, without subgroup analysis, reported an association between current smoking status and the risk of HUA in women [5,6], which is consistent with our findings. However, in our subgroup analysis, current smoking habits were not associated with increased risk of HUA in women without MetS. In women with MetS, a smoking habit was associated with the risk of HUA. Our results suggest that the strong impact of current smoking on HUA in women may be attributed to the confounding effect of MetS. Women who currently smoke are more likely to have MetS [46] because smoking is associated with metabolic risk factors such as insulin resistance, abdominal obesity, and dyslipidemia, which are components of MetS [47]. This subsequently increases their susceptibility to HUA. One notable discovery in this study is that women who have both MetS and are current smokers face a significantly higher risk of developing HUA, up to four times greater than the reference group. This finding suggests that although MetS may play a more dominant role than smoking in the development of HUA, the combination of MetS and current smoking among women may have a synergistic effect on HUA development. MetS and current smoking habits are all modifiable factors through lifestyle changes and/or medical treatment. Although this result does not imply causality, careful monitoring and management may be necessary for the prevention of HUA in women smokers with MetS. Considering previous results that indicate a 50% reduction in the risk of developing gout when recovering from MetS, treating MetS could serve as secondary prevention for HUA [48]. In a UK biobank study, it was confirmed that adopting a healthy lifestyle can reduce the risk of gout associated with genetic factors by almost one-third. The importance of correcting MetS and unhealthy lifestyle habits is evident [26].

In men, these relationships were more pronounced in non-smokers, unlike the findings in women. Further research will be needed to elucidate the mechanism underlying these sex differences. Through subgroup analysis based on alcohol consumption and the presence of MetS, we confirmed that women are more vulnerable to MetS and unhealthy lifestyle behaviors.

The relationship between HTN and HUA has previously been established. Previous studies have mainly focused on the association between HUA and incident HTN, but the results have been inconsistent with respect to sex effects [49,50,51,52]. Our study identified a significant association between HTN and an increased risk of HUA only in women. In our study, we also discovered a variation in the association between DM and HUA according to sex. Specifically, among women, having DM was linked to an increased risk of HUA. However, interestingly, diabetic males were found to have a negative association with HUA. These observations align with the results reported in previous research studies that support similar conclusions [38,53,54]. Hyperglycemia can cause alterations in uric acid reabsorption and excretion in the proximal tubules, and insulin resistance can also lead to tubular dysfunction. Presumably, the effects of insulin resistance and glucose metabolism on urate metabolism are expected to differ between men and women [55]. However, the exact mechanism for this has not been clarified. Also, lifestyle, treatment compliance, hormonal effects, sex-based differences in body fat composition, and comorbidities are possible mechanisms.

There are some limitations in our research. This study was conducted as a cross-sectional study, and even if the association between HUA and potential risk factors was confirmed, a causal relationship cannot be established. Second, since KNHANES did not include detailed information about underlying diseases or medications taken by subjects that affect serum urate, we could not adjust for medication or diagnosis of gout in analyses. Furthermore, the findings are not generalizable beyond Korean individuals. This research, however, confirms the sex-specific link between alterable risk factors and HUA. Additionally, this study identifies a subgroup with an extremely high risk of developing HUA. These findings have important implications for the creation of clinical guidelines that are tailored to specific sexes.

In conclusion, MetS has been determined to be a significant risk factor for HUA in both men and women, regardless of lifestyle behaviors. The influence of lifestyle behaviors on HUA varied depending on the presence of MetS. These associations varied by gender. The presence of MetS coupled with unhealthy habits amplifies the susceptibility to developing HUA particularly among women. Sex-specific screening measures aimed at identifying asymptomatic HUA cases along with preventive strategies to mitigate potential complications and alleviate socio-economic burdens are necessary. This study’s findings suggest the need for special attention to HUA in women with MetS who have unhealthy lifestyle habits (current smoker, heavy drinker). Additional clinical trials involving interventions related to lifestyle habits and MetS are required to ascertain the causality of the observed associations.

## Figures and Tables

**Table 1:** Baseline characteristics of study population according to sex (N = 16,288).

	Male (N = 7154)	Female (N = 9134)	*p* Value
Uric acid (mg/dL)	5.95 ± 0.02	4.41 ± 0.01	<0.0001
Hyperuricemia, %	21.06 (0.63)	6.61 (0.31)	<0.0001
Age (years)	45.76 ± 0.27	47.75 ± 0.28	<0.0001
Level of education, %			<0.0001
Elementary school graduate or less	8.77 (0.4)	18.67 (0.63)	
Middle school graduate	8.21 (0.41)	9.08 (0.37)	
High school graduate	37.17 (0.78)	33.88 (0.69)	
University graduate or more	45.85 (0.96)	38.36 (0.84)	
Level of income, %			<0.0001
Q1	13.03 (0.6)	16.78 (0.65)	
Q2	22.64 (0.69)	23.98 (0.65)	
Q3	30.97 (0.74)	28.97 (0.67)	
Q4	33.36 (0.96)	30.28 (0.89)	
Occupation, yes, %	76.53 (0.66)	54.64 (0.71)	<0.0001
Spouse, yes, %	93.09 (0.43)	80.56 (0.62)	<0.0001
Smoking, yes, %	37.06 (0.77)	5.66 (0.33)	<0.0001
Amount of alcohol intake			<0.0001
None	13.8 (0.51)	30.24 (0.61)	
Mild	70.19 (0.66)	66.92 (0.62)	
Heavy	16 (0.52)	2.84 (0.23)	
Physically active, %	50.23 (0.79)	43.56 (0.7)	<0.0001
Diabetes, %	11.64 (0.44)	8.99 (0.38)	<0.0001
Hypertension, %	30.66 (0.66)	23.65 (0.63)	<0.0001
Hypercholesterolemia, %	18.66 (0.55)	21.58 (0.49)	<0.0001
Chronic kidney disease, %	3.08 (0.21)	2.95 (0.19)	0.6212
BMI (Kg/m^2^)	24.58 ± 0.05	23.32 ± 0.05	<0.0001
WC (cm)	86.21 ± 0.14	78.18 ± 0.16	<0.0001
Total cholesterol (mg/dL)	192.08 ± 0.55	193.8 ± 0.46	0.0084
HDL-C (mg/dL)	47.45 ± 0.16	55.04 ± 0.18	<0.0001
Fasting plasma glucose (mg/dL)	102.15 ± 0.37	97.14 ± 0.27	<0.0001
Systolic BP (mmHg)	119.89 ± 0.23	115 ± 0.26	<0.0001
Diastolic BP (mmHg)	78.47 ± 0.16	73.48 ± 0.14	<0.0001
Metabolic syndrome, yes, %	34.45 (0.69)	26.13 (0.62)	<0.0001
BMI			<0.0001
<23	32.81 (0.66)	52.27 (0.69)	
≥23, <25	25.3 (0.61)	19.75(0.47)	
≥25	41.89 (0.71)	27.98 (0.61)	
Eating alone (times/day)			<0.0001
0 times	51.96 (0.88)	40.64 (0.7)	
1 time/day	29.42 (0.71)	33.47 (0.63)	
2 times /day	13.01 (0.53)	17.81 (0.5)	
3 times/day	5.61 (0.35)	8.08 (0.35)	
Menopause, yes		40.1 (0.78)	

**Table 2:** The association of lifestyle factors with the risk of hyperuricemia according to sex.

	Male			Female		
	Univariate	Model 1	Model 2	Univariate	Model 1	Model 2
Smoking						
Non smoker	1 (ref.)	1 (ref.)	1 (ref.)	1 (ref.)	1 (ref.)	1 (ref.)
Ex-smoker	0.82 (0.69, 0.98)	1.16 (0.91, 1.47)	1.13 (0.88, 1.44)	0.96 (0.62, 1.49)	1.06 (0.62, 1.80)	0.97 (0.55, 1.73)
Current smoker	0.95 (0.81, 1.12)	1.02 (0.80, 1.31)	1.03 (0.80, 1.32)	1.61 (1.14, 2.28)	1.60 (1.02, 2.53)	1.60 (1.01, 2.53)
Alcohol						
None	1 (ref.)	1 (ref.)	1 (ref.)	1 (ref.)	1 (ref.)	1 (ref.)
Mild	1.41 (1.14, 1.75)	1.13 (0.88, 1.44)	1.21 (0.93, 1.56)	0.78 (0.65, 0.94)	1.16 (0.92, 1.46)	1.34 (1.04, 1.72)
Heavy	1.79 (1.39, 2.32)	1.5 (1.10, 2.05)	1.50 (1.08, 2.08)	1.41 (0.88, 2.26)	1.79 (0.92, 3.48)	1.68 (0.85, 3.29)
Physically active exercise						
No	1 (ref.)	1 (ref.)	1 (ref.)	1 (ref.)	1 (ref.)	1 (ref.)
Yes	1.166 (1.01, 1.34)	1.038 (0.86, 1.26)	1.072 (0.88, 1.31)	0.928 (0.77, 1.11)	0.945 (0.75, 1.18)	1.064 (0.84, 1.35)

Model 1: Age, level of education, income, occupation, spouse, smoking, alcohol intake, physically active exercise, eating alone. Model 2: Age, level of education, income, occupation, spouse, smoking, alcohol intake, physically active exercise, eating alone, BMI, MetS, CKD.

**Table 3:** The association of metabolic syndrome and its parameters with the risk of hyperuricemia according to sex.

	Male			Female		
	Univariate	Model 1	Model 2	Univariate	Model 1	Model 2
Metabolic Syndrome						
No	1 (ref.)	1 (ref.)	1 (ref.)	1 (ref.)	1 (ref.)	1 (ref.)
Yes	1.87 (1.63, 2.14)	2.26 (1.88, 2.72)	1.80 (1.47, 2.21)	4.04 (3.31, 4.93)	3.66 (2.82, 4.75)	2.40 (1.78, 3.23)
Metabolic Components, Yes vs. No (Reference)						
Raised WC	2.06 (1.81, 2.34)	1.79 (1.50, 2.13)	1.23 (0.99, 1.54)	4.26 (3.51, 5.18)	3.33 (2.63, 4.22)	1.78 (1.31, 2.42)
Elevated TG	1.81 (1.59, 2.06)	2.13 (1.78, 2.55)	1.84 (1.52, 2.21)	2.88 (2.36, 3.50)	2.27 (1.78, 2.89)	1.81 (1.41, 2.34)
Hyperglycemia	1.02 (0.89, 1.17)	1.16 (0.97, 1.39)	0.97 (0.81, 1.17)	2.66 (2.20, 3.22)	2.11 (1.569, 2.63)	1.61 (1.26, 2.05)
Elevated BP	1.54 (1.35, 1.75)	2.10 (1.71, 2.58)	1.73 (1.39, 2.15)	2.86 (2.33, 3.50)	2.70 (2.01, 3.62)	2.11 (1.56, 2.86)
Low HDL	2.86 (2.33, 3.50)	2.70 (2.01, 3.62)	2.11 (1.56, 2.86)	2.30 (1.90, 2.79)	1.94 (1.52, 2.47)	1.53 (1.19, 1.97)
Number of Metabolic components						
0	1 (ref.)	1 (ref.)	1 (ref.)	1 (ref.)	1 (ref.)	1 (ref.)
1	1.42 (1.13, 1.79)	1.40 (0.99, 1.99)	1.23 (0.86, 1.77)	1.37 (0.92, 2.02)	1.38 (0.80, 2.36)	1.12 (0.64, 1.95)
2	1.70 (1.36, 2.12)	2.09 (1.51, 2.88)	1.74 (1.24, 2.43)	3.80 (2.64, 5.46)	4.44 (2.65, 7.43)	3.15 (1.82, 5.44)
3	2.36 (1.89, 2.95)	2.94 (2.09, 4.14)	2.28 (1.57, 3.30)	5.64 (3.97, 8.01)	6.87 (4.22, 11.18)	4.35 (2.52, 7.50)
≥4	2.71 (2.19, 3.35)	4.1 (2.96, 5.68)	2.90 (2.01, 4.18)	7.76 (5.64, 10.68)	9.02 (5.63, 14.45)	4.80 (2.77, 8.30)

Model 1: Age, level of education, income, occupation, spouse, smoking, alcohol intake, physically active exercise, eating alone. Model 2: Age, level of education, income, occupation, spouse, smoking, alcohol intake, physically active exercise, eating alone, BMI, CKD.

**Table 4:** The association of major cardiovascular risk factors with the risk of hyperuricemia.

	Male			Female		
	Univariate	Model 1	Model 2	Univariate	Model 1	Model 2
Hypertension	1.28 (1.11, 1.49)	1.79 (1.45, 2.20)	1.19 (0.93, 1.51)	3.10 (2.55, 3.77)	2.81 (2.13, 3.71)	1.7 (1.27, 2.29)
Diabetes	0.62 (0.49, 0.77)	0.76 (0.59, 0.99)	0.48 (0.36, 0.65)	2.62 (2.05, 3.36)	1.91 (1.45, 2.52)	0.98 (0.71, 1.34)
Hypercholesterolema	1.12 (0.95, 1.33)	1.36 (1.11, 1.66)	1.01 (0.81, 1.26)	2.04 (1.67, 2.48)	1.68 (1.31, 2.14)	1.33 (0.99, 1.76)

Model 1: Age, level of education, income, occupation, spouse, smoking, alcohol intake, physically active exercise, eating alone. Model 2: Age, level of education, income, occupation, spouse, smoking, alcohol intake, physically active exercise, eating alone, MetS, BMI, CKD.

**Table 5:** The risk of hyperuricemia according to the presence of metabolic syndrome and smoking status.

	Male			Female		
	Univariate	Model 1	Model 2	Univariate	Model 1	Model 2
Metabolic Syndrome, No						
Non-smoker	1 (ref.)	1 (ref.)	1 (ref.)	1 (ref.)	1 (ref.)	1 (ref.)
Ex-smoker	0.83 (0.65, 1.05)	1.57 (1.10, 2.23)	1.56 (1.10, 2.21)	1.3 (0.71, 2.37)	1.48 (0.71, 3.10)	1.36 (0.64, 2.91)
Current smoker	0.99 (0.80,1.22)	1.30 (0.91, 1.87)	1.34 (0.94, 1.92)	1.86 (1.11, 3.14)	1.36 (0.60, 3.05)	1.41 (0.61, 3.27)
Metabolic Syndrome, Yes						
Non-smoker	2.39 (1.87, 3.06)	3.68 (2.52, 5.39)	2.89 (1.93, 4.33)	4.20 (3.40, 5.19)	3.72 (2.83, 4.89)	2.44 (1.77, 3.35)
Ex-smoker	1.47 (1.17, 1.84)	2.99 (2.11, 4.25)	2.35 (1.63, 3.40)	3.49 (1.79, 6.80)	2.59 (1.20, 5.62)	1.60 (0.69, 3.68)
Current smoker	1.74 (1.38, 2.20)	2.68 (1.84, 3.90)	2.23 (1.51, 3.30)	7.01 (4.49, 10.95)	6.27 (3.69, 10.64)	4.26 (2.47, 7.35)

Model 1: Age, level of education, income, occupation, spouse, smoking, alcohol intake, physically active exercise, eating alone. Model 2: Age, level of education, income, occupation, spouse, smoking, alcohol intake, physically active exercise, eating alone, BMI, CKD.

**Table 6:** The risk of hyperuricemia according to the presence of metabolic syndrome and alcohol intake.

	Male			Female		
	Univariate	Model 1	Model 2	Univariate	Model 1	Model 2
Metabolic Syndrome, No						
Non-drinker	1 (ref.)	1 (ref.)	1 (ref.)	1 (ref.)	1 (ref.)	1 (ref.)
Mild drinker	1.66 (1.21, 2.28)	1.29 (0.89, 1.86)	1.31 (0.91, 1.89)	1.07 (0.76, 1.49)	1.15 (0.75, 1.74)	1.22 (0.80, 1.88)
Heavy drinker	2.27 (1.55, 3.33)	1.71 (1.04, 2.81)	1.71 (1.03, 2.84)	2.19 (1.09, 4.39)	1.50 (0.51, 4.39)	1.37 (0.46, 4.10)
Metabolic Syndrome, Yes						
Non-drinker	2.42 (1.64, 3.57)	2.99 (1.9, 4.69)	2.10 (1.30, 3.38)	4.19 (3.01, 5.84)	3.51 (2.38, 5.17)	2.18 (1.42, 3.35)
Mild drinker	3.19 (2.30, 4.44)	2.90 (1.96, 4.25)	2.35 (1.57, 3.50)	4.43 (3.20, 6.13)	4.29 (2.93, 6.28)	3.06 (2.05, 4.57)
Heavy drinker	3.18 (2.18, 4.65)	3.27 (2.08, 5.13)	2.83 (1.78, 4.5)	6.79 (3.18, 14.5)	5.71 (2.34, 13.9)	4.13 (1.68, 10.2)

Model 1: Age, level of education, income, occupation, spouse, smoking, alcohol intake, physically active exercise, eating alone. Model 2: Age, level of education, income, occupation, spouse, smoking, alcohol intake, physically active exercise, eating alone, BMI, CKD.

## Data Availability

The data presented in this study are available on request from the corresponding author.
